# Mechanical Properties of PUR and Latex Foams as Predictors for Seating or Lying Comfort

**DOI:** 10.3390/polym18121549

**Published:** 2026-06-22

**Authors:** Zoran Vlaović, Danijela Domljan, Tomislav Gržan, Goran Mihulja

**Affiliations:** Department of Furniture and Wood in Construction, Faculty of Forestry and Wood Technology, University of Zagreb, 10000 Zagreb, Croatia; zvlaovic@sumfak.unizg.hr (Z.V.); ddomljan@sumfak.unizg.hr (D.D.); gmihulja@sumfak.unizg.hr (G.M.)

**Keywords:** multilayer structures, polyurethane and latex foams, compression modulus, recovery, hysteresis loss, comfort, sitting, lying

## Abstract

Flexible polyurethane (PUR) foams and latex rubber foams are widely used in furniture and mattress cushioning, yet conventional standardized mechanical tests only partially capture comfort-relevant behavior, particularly in layered constructions where material interactions and sequencing can alter elastic response. This study aimed to compare the mechanical (elastic) properties of selected three-layer composites of approximately 60 mm thickness (composed of conventional PUR, high-resilience PUR, low-resilience PUR, and latex foam) and to preliminarily assess whether combining foam types improves support of such setup and whether changing layer order modifies elasticity and support. Indentation hardness testing of multilayer cushions was conducted by ISO 2439:2008 Method E. Six three-layer systems (Alpha–Zeta) were assembled in two groups. Group X showed nearly identical support factors (2.6–2.7), high recovery (64.3–66.2%), low hysteresis loss (24.3–24.5%), and overlapping force–indentation (IFD) curves, indicating minimal effect of layer order and dominance of the PUR layers. Group Y exhibited higher but more sequence-dependent support (3.1–3.7), markedly reduced, wider range recovery (30.0–45.9%), increased hysteresis (33.0–34.7%), and more dispersed IFD curves. Placing high-resilience foam at the top partially improve recovery, whereas locating low-resilience foam at the surface increase energy loss. The research contributes in part to the body of knowledge about the behavior of the tested materials according to standardized rules. These preliminary results can be compared with other research findings and used in the preparation of testing models for multilayer foam composites, thereby generating new knowledge to improve the design of future experiments, which will result in increased sitting and lying comfort.

## 1. Introduction

Today, flexible polyurethane (PUR) foams are commonly used in the manufacture of furniture and mattresses. These foams are known for their ability to provide comfort, support, and durability—all essential for applications in seats and beds. Flexible foams range from conventional open-cell foams, through high-resilience (HR) and low-resilience (LR) foams to hybrid types such as latex-infused foams. Besides PUR materials, latex rubber foam is also frequently used in furniture. This study investigated the elastic properties of certain multilayer composites of PUR foams of various types and one latex foam. Evaluations of the mechanical properties of flexible PUR foams are carried out by testing using standardized methods, such as ISO 2439 [1], ISO 3386-1 [2] or ASTM D3574 [3]. Key properties such as density, indentation hardness (IFD/ILD), compression modulus (support factor), hysteresis (energy) loss, recovery behavior, compression stress/strain characteristics, etc., provide a basic framework for comparing material formulations. However, the results of mechanical tests alone cannot capture and interpret possible user experiences. Comfort, in particular, is shaped by multiple factors beyond stiffness or energy return, and its assessment remains partly subjective [4]. The development of comfort-oriented products often involves a duality between the objectivity of experimental data, mechanical tests and models and the subjectivity of experiences (feelings of comfort), and this is often shaped by different norms and standards. Researchers often use dual assessment frameworks. Studies have used material compression tests alongside seating comfort tests to jointly quantify ergonomic and mechanical performance, or correlate physical variables (contact pressure, weight distribution, skin temperature at the contact surface) [5,6] with perceived comfort of subjective comfort ratings to optimize product design [7]. In addition, virtual prototyping, finite element models, or direct (numerical) parameter identification methods [8] are often used to develop correlated numerical models and proactively assess perceived comfort, which can then be tested experimentally [5,6].

The researchers used finite element method and analysis (FEM/FEA) in their papers, which is considered to be a reliable tool for characterizing monolithic and composite PUR foams—especially for validating mechanical properties and modeling viscoelastic responses and large deformations [5,9,10]. However, its reliability is challenged and limited by the complexity of the foam micromechanics, the nonlinear nature of its properties, the need for empirical data to characterize the behavior at high stresses, and the computational constrains [11,12].

Such holistic approaches, guided by standards and new design guidelines focused on comfort, are key to innovation and to the creation of products that can improve user comfort, despite the inherent challenges posed by the specific variability and complexity of human–product interaction.

Density and firmness are key parameters influencing the comfort and support characteristics of flexible PUR foams. These two properties function independently and must be carefully balanced in product design. While increased density generally correlates with a higher compressive modulus—indicating the foam’s greater resistance to deformation under load—this relationship does not always translate directly into improved comfort. In furniture applications, higher-density foams are often preferred for their structural integrity and load-bearing capacity [13,14]. In some cases, latex rubber foam, due to its inherently higher density, may outperform conventional PUR foams in terms of support; however, PUR foams can also surpass latex in properties such as IFD recovery [4].

A property that captures long-term deformation under load is the compression set, defined as the foam’s ability to recover its original thickness following prolonged compression. This metric is particularly sensitive to density, as higher-density foams typically exhibit lower values of compression set, suggesting better recovery and reduced permanent deformation [15]. In viscoelastic formulations, performance becomes even more dependent on external factors. These foams are known to stiffen and exhibit greater hysteresis under faster strain rates and lower ambient temperatures [13,14,16,17].

Low-resilience (viscoelastic) foams are distinguished by their slow recovery characteristics and high damping behavior, often exhibiting greater hysteresis and lower resilience compared to HR foams. Their ability to precisely adapt to body contours, makes them suitable for applications requiring pressure distribution and improved support [18,19]. In contrast, HR foams are noted for maintaining relatively stable performance across varying conditions [20]. Improved resilience and energy return make HR foams well-suited for settings where consistent support and long-term durability are priorities [18]. Latex foams, by comparison, have demonstrated higher ball-rebound heights, suggesting enhanced elastic response (or “bounce-back”) when measured against conventional PUR and LR foams. While this may indicate superior energy return, such metrics must be interpreted in context, as they do not directly reflect user-perceived comfort under sustained loads.

Foam thickness is also important in mechanical performance, especially in layered or seating configurations. Thicker foam layers have been shown to resist deformation at 25% compression more effectively, reflecting higher stiffness. On the other hand, very thin foams can lead to higher interface pressures and an increased risk of bottoming out, potentially reducing comfort [21]. Thus, the thickness needs to be optimized in relation to stiffness and pressure distribution. The last characteristic in this preview is hysteresis loss—lower hysteresis loss implies more efficient energy return, which may enhance both comfort and the long-term resilience of the foam [21].

Layered cushion constructions appear to differ significantly from single-layer systems, both in their mechanical response and in how users perceive comfort. In multilayer PUR foam cushions, the selection and sequence of foams of different hardness and elastic behavior play a significant role in long-term durability and resistance to mechanical fatigue. For example, Silva et al. reported that incorporating higher-density foams into layered configurations was associated with improved static load performance and with higher perceived comfort [21].

A recurring design logic is to place softer foams near the human body, while relying on firmer layers beneath to limit deep sink-in. This arrangement is often presented as a compromise between comfort and postural support. That is assumed to be important for limiting localized tissue loading, which may help reduce discomfort and the risk of pressure-related skin damage during long-term sitting [22,23]. Still, the balance is not straightforward. Liu, Siridhar and Wang suggest that when overall cushion thickness must be reduced (a common constraint in contemporary chair designs), harder foams may be necessary to preserve adequate support [24]. At the same time, excessively stiff materials have repeatedly been linked to the formation of pressure concentrations and to diminished sitting comfort [21,25].

The issue becomes even less clear-cut in zoned multilayer systems, where different regions of the cushion are intentionally tuned to support specific anatomical areas. In such designs, the relationship between density and perceived firmness no longer follows a simple linear pattern. Instead, the compressive response of the cushion appears to emerge from a more intricate interplay between foam formulation and external factors, including temperature and loading rate, both of which are known to alter the viscoelastic behavior of PUR materials.

User-specific factors should not be neglected in cushion design. Foam elasticity affects the pressure distribution and shape retention. High elasticity enhances durability by reducing deformation and sagging [18,23]. Moon et al. explore how PUR materials affect seat cushion comfort, using a combination of polyurethane foam, viscoelastic foam, and Technogel^®^ in multilayer cushions [22]. The research shows that hardness, support factor, hysteresis loss, and a few other characteristics are key parameters from IFD curves for assessing short-term comfort. Viscoelastic foam and Technogel^®^ in different layers affect the softness and support properties. Technogel^®^ enhances adaptive support by behaving like a fluid during deformation and distributing pressure evenly. The gaps in the existing research on the mechanical testing of layered PUR foam systems include the need for more accurate models that integrate physical testing data and improved theoretical frameworks that account for the material distribution within the foam structure. Physical testing is needed to determine the mechanical properties of multi-layered foam materials under realistic conditions and to validate models that accommodate different indenter sizes and shapes for real-world sitting conditions, matching cushion properties to individual needs [23,26,27].

We believe that numerical approaches, such as finite element modeling, can provide valuable complementary insight into the deformation behavior of multilayer foam systems, particularly in relation to stress distribution, local deformation, and load transfer between layers. However, the present study was designed as a preliminary experimental investigation focused on the standardized mechanical response of selected PUR and latex foam composites under indentation loading. A reliable FEM/FEA model of such systems would require nonlinear and viscoelastic material characterization of each individual foam layer, as well as the definition of layer-to-layer interactions. This aspect was not omitted as an oversight, but was deliberately excluded to maintain a focused interpretation of the investigated variables.

The aim of this study was to experimentally research and compare the mechanical properties of latex and PUR foams of different types and their layered combinations, but not to develop a numerical model. As a preliminary paper, this research represents an investigation for future multilayer structures that will have more favorable elastic properties, provide better support, and consequently greater comfort for sitting or lying. Two hypotheses have been proposed: first, that combining different foam types can yield better support properties, and, second, that changing the order of the foam types in the layers can influence the elasticity and support properties of the resulting composite.

## 2. Materials and Methods

This research included selected flexible PUR foams (conventional, HR, and LR) and latex rubber foam, obtained from several manufacturers in Southeast and Central Europe. The samples were new and had not been previously used.

### 2.1. Foam Samples

**Figure 1:**
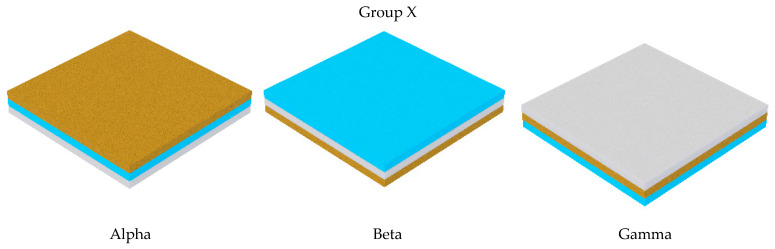

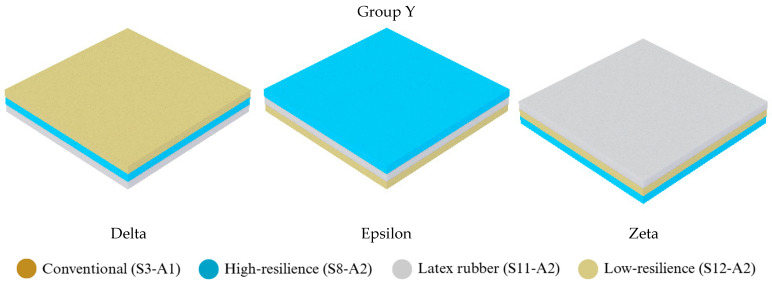
Sample compositions, groups and samples markings.

The samples used in this study consisted of six multilayer foam systems, labeled Alpha to Zeta. Each system was composed of three individual layers (sub-samples), selected amongst four different foam types. The multilayer systems were classified into two groups, Group X and Group Y, based solely on the type of polyurethane foam used (Figure 1).

Both groups contained the sub-samples S8 and S11, and differed only in the third foam layer: Group X included a conventional foam (S3), whereas Group Y included a low-resilience foam (S12). Within each group, the individual multilayer systems differed only in the stacking sequence of the three layers, while the overall structural concept remained the same.

All sub-samples were in the form of square plates measuring (390 × 390) mm with a thickness of approximately 20 mm (Table 1). They were used to fabricate multilayer cushions approximately 60 mm thick by stacking components of different compositions as shown in Figure 1.

For the purposes of this paper, Table 1 presents basic data on the foams used as sub-samples, while other data, including indentation hardness values and detailed measurements, are available in our previous work [29].

The sub-samples were previously conditioned under laboratory climate air conditions of (23 ± 2) °C and (50 ± 5)% relative humidity per the requirements of ISO 845:2006 [28] and ISO 2439:2008 [1].

### 2.2. Methods

**Figure 2:**
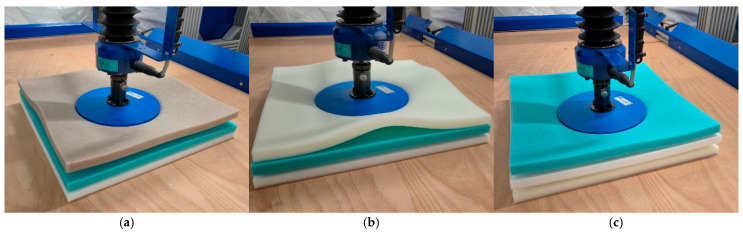
Inspekt S—pressing an indenter into the sample: (**a**) Alpha; (**b**) Delta; (**c**) Epsilon (photo: ©Vlaović).

After conditioning, the sub-samples were used to determine the apparent core density of cellular plastics and rubbers in accordance with ISO 845:2006 [28]. Individual layers were weighed on an analytical balance (Sartorius ED224S-0CE, Sartorius Corp., Edgewood, NY, USA), weighing range 0–220 g, readability 0.1 mg) and the foam density was calculated (Table 1). The thickness of the sub-samples, as well as their elastic characteristics, was measured using a universal testing machine Inspekt S (load range 0–2 kN, LabMaster ver. 3.0.5.26; H&P MPT GmbH, Dresden, Germany), using a circular indenter (diameter 200 mm) under a preload force of 4.8–5.0 N (Figure 2).

Subsequently, indentation hardness testing was conducted on the multilayer cushions, and the support factor (Sf—compressive deflection coefficient), recovery (hysteresis return), and hysteresis loss rate (Af) were calculated according to Method E in ISO 2439:2008 [1]. The measurements were repeated three times for each sample (on same sample after at least 16 h) and the data presented for each sample corresponding to the average values.

For the purposes of this research, the sub-samples were not glued when testing their different combinations, due to the limited number of available sheets. This was the only deviation from ISO 2439:2008 [1] during the experimental procedure. The standardized indentation hardness test method is described in detail in the respective ISO standard.

## 3. Results and Discussion

This chapter presents results related to the investigation of the mechanical (elastic) properties of multilayer samples. It is important to note that the values shown for individual foam layers (sub-samples S3, S8, S11 and S12) are the results of previous tests and are not variable in these multilayer systems. These values represent the intrinsic properties of each layer and are used to compare and interpret the results obtained for samples Alpha to Zeta.

### 3.1. Results of Support Factor

**Figure 3:**
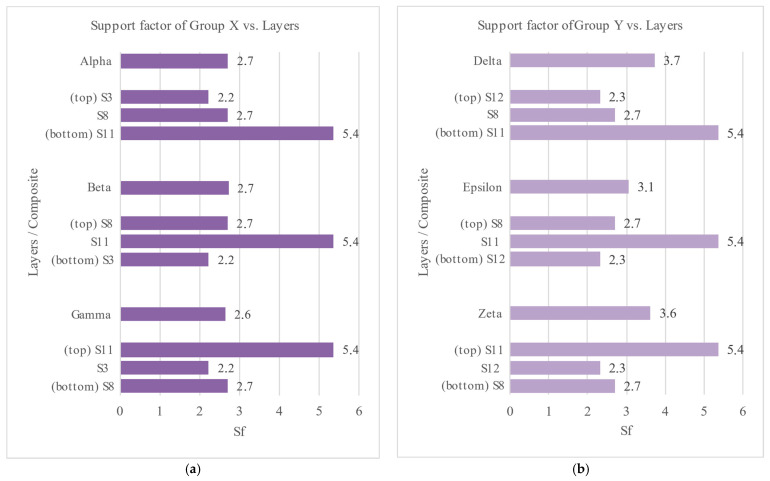
Dependence of support factor on the arrangement of layers in a particular composite (the bigger, the better): (**a**) in Group X; (**b**) in Group Y.

A compressive deflection coefficient (Sf) (Figure 3) accounts for the foam’s ability to retain support under stress, which is more relevant to fatigue, durability, and hysteresis effects. Here, the tests provided through Method E are relevant for assessing long-term support, particularly in applications where the foam is repeatedly stressed and expected to recover. Similar IFD-derived parameters have been used to evaluate the comfort-related behavior of multilayer polyurethane cushions. At the same time, pressure-distribution studies further show that layered foam performance depends on foam configuration and loading conditions [6,7,22].

Group X composites (samples Alpha, Beta, Gamma) exhibit nearly identical global support factors (2.6–2.7), despite different stacking sequences. This demonstrates that, within a system containing S3, S8 and S11, the stacking order has negligible influence on the global support factor. Despite S11 having an exceptionally high inherent support factor (5.4), incorporating it into the composite structure does not substantially elevate the overall support factor above roughly 2.7. Rather, the composite behavior aligns with the characteristics of the softer polyurethane layers (with S3 at 2.2 and S8 at 2.7). This suggests that the less-rigid layers govern the overall mechanical performance, effectively constraining the extent to which the high-support latex rubber foam influences the system’s global characteristics. Consequently, in Group X, the choice of materials has a greater impact than the arrangement of layers, and simply adding a high-support layer is not enough to produce a significant increase in total support.

Group Y composites show consistently higher support factors (3.1–3.7) and a wider dispersion compared with Group X. Since the intrinsic properties of S8 and S11 are identical in both groups, the increase can be attributed directly to the substitution of S3 by S12. Although the support factor of S12 is only marginally different from S3 at the single-layer level (2.3 vs. 2.2), this substitution produces a substantial increase in composite support. This demonstrates that small differences in intrinsic foam properties can be amplified in multilayer systems, particularly when they affect the compliance of the softest layer in the stack. Furthermore, the wider range of support factors in Group Y indicates that layer order becomes more influential once the softest layer is moderately improved. This is consistent with the broader concept that multilayer cushion performance results from the combined action of layer properties, layer positions and loading conditions, rather than from the behavior of a single component [6,22].

### 3.2. Results of Recovery

**Figure 4:**
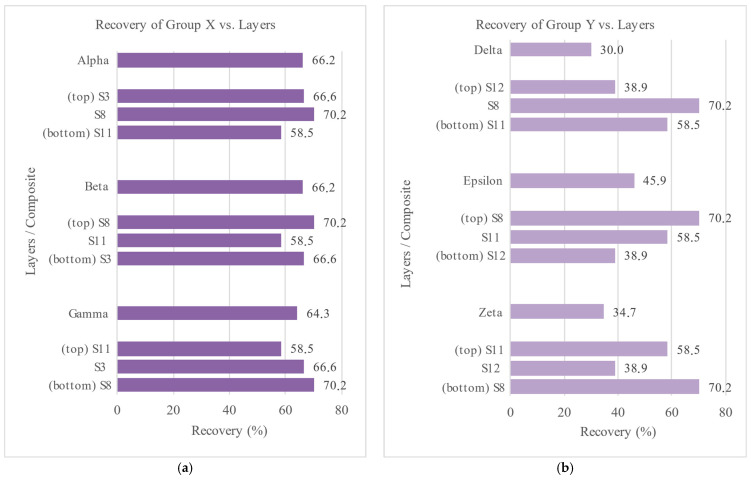
Dependence of recovery (at 25% IFD) on the arrangement of layers (the bigger, the better): (**a**) in Group X; (**b**) in Group Y.

A hysteresis return, known as recovery, in this case measured at surface level on 25% IFD, directly relates to the energy loss during deformation, and is a measure of how well a flexible PUR foam regains its original thickness and properties after being deformed and unloaded (Figure 4). The greater the recovery, the lower the hysteresis loss, meaning the foam “springs back” more faithfully to its original shape [30]. Recovery and hysteresis are commonly interpreted as complementary indicators of elastic return and energy dissipation in flexible foam systems, and both have been linked to comfort-related mechanical behavior in previous studies [10,11,22].

Group X exhibits high, narrowly distributed recovery values (64.3–66.2%), indicating that the multilayer systems retain a fast and stable elastic response irrespective of layer sequencing. The overall recovery performance of the composite structures closely mirrors that of the PUR foam layers (S3 and S8) and consistently exceeds that of the latex foam (S11). Therefore, despite the presence of latex in each configuration, its reduced recovery capacity does not affect the overall recovery performance of the observed composites. Rather, the multilayer system’s behavior tends toward the better recovery characteristics exhibited by the PUR components. In fact, positioning the lower-recovery latex layer on top produces only a modest reduction in global recovery. Group X illustrates that PUR foams with high recovery properties maintain the stability of the multilayer system’s behavior, thereby placing the ordering of layers, aimed at increasing recovery performance, in the background. Group Y composites (Delta, Epsilon and Zeta) exhibit reduced and quite scattered recovery values (30.0–45.9%). The introduction of low-resilience foam obviously reduces the composite recovery. Even though S8 remains present across all configurations, the global recovery leans toward values of S12. The recovery values of the composites fall between those of the latex and viscoelastic foams, indicating that the low-resilience foam governs the dynamic response of the multilayer system. Layer sequencing has a pronounced effect. Epsilon (45.9%), with S8 at the top, shows the highest recovery, whereas Delta (30.0%), with visco-foam at the top, exhibits the lowest. This indicates that the surface layer strongly influences perceived and measured recovery, and that positioning a high-resilience foam at the top can partially mitigate, but not overcome, the damping effect of low-resilience foam.

### 3.3. Results of Hysteresis Loss Rate

**Figure 5:**
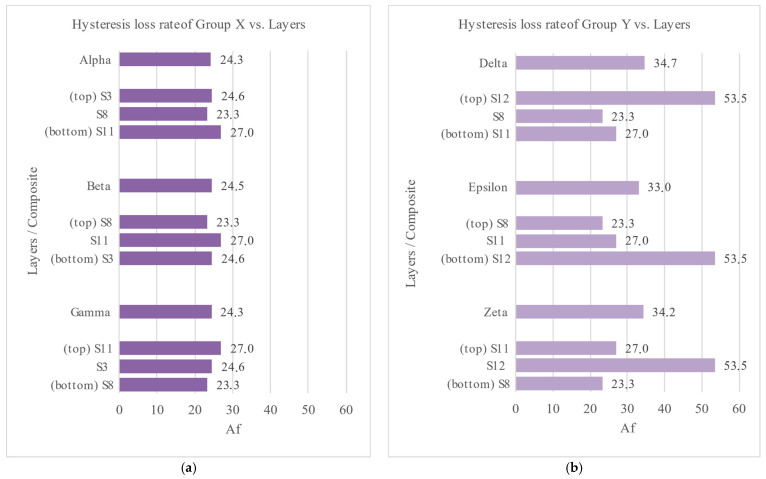
Dependence of hysteresis loss rate on the arrangement of layers (the less, the better): (**a**) in Group X; (**b**) in Group Y.

A hysteresis loss rate (Af), expressed as a percentage of the loading energy, represents the energy difference between the loading and unloading of a test piece under cyclic deformation (Figure 5)—providing additional information about materials’ load-bearing properties. The interpretation of hysteresis as an energy-dissipation response is supported by studies describing flexible polyurethane foams through nonlinear elastic and viscoelastic contributions, with mechanical losses affected by molecular mobility, chemical structure and cyclic loading behavior [10,11,12].

The hysteresis loss rate of Group X composites is quite uniform, with values ranging narrowly between 24.3 and 24.5%. This very small dispersion indicates that layer sequencing has practically no influence on the energy dissipation behavior of the multilayer cushions when composed of S3, S8 and S11 foams. Despite the presence of latex foam (S11), which has the highest intrinsic hysteresis, the composite Af does not approach 27% but instead stabilizes near the hysteresis levels of the PUR foams. This demonstrates that energy dissipation in the multilayer system is governed primarily by the lower-loss PUR layers, which effectively limit the contribution of the more dissipative latex foam. Minor differences in hysteresis loss between Alpha (24.3%), Beta (24.5%), and Gamma (24.3%) are within experimental scatter and confirm that positioning of the latex foam layer (top, middle, or bottom) does not meaningfully affect overall hysteresis behavior. In other words, Group X multilayer cushions show stable, low-to-moderate hysteresis, consistent with their high recovery and moderate support factors. The data indicate that the viscoelastic damping of the system is dominated by the PUR layers, making Group X configurations mechanically robust to changes in stacking sequence.

The hysteresis loss rate of Group Y composites exhibits substantially higher hysteresis loss rates than Group X, with values ranging from 33.0 to 34.7%. This indicates a pronounced increase in energy dissipation and internal damping. The elevated composite hysteresis loss rate values arise directly from the introduction of LR foam (S12). Although S12 has a very high intrinsic hysteresis loss (53.5%), the composite hysteresis loss does not approach this value, but instead stabilizes around 33–35%. This demonstrates that the low capacity of LR foam is partially constrained by the lower-loss S8 and S11 layers yet still dominates the overall viscoelastic response. Unlike Group X, layer sequencing has a measurable influence. Situations where low-resilience foam is placed at the top show the highest hysteresis; whereas, those where the LR foam layer is at the bottom show the lowest. This indicates that placing a highly dissipative layer closer to the load-application surface increases global energy loss, whereas positioning it deeper in the stack partially mitigates it through the less dissipative layers above.

### 3.4. Force–Indentation Curves of Multilayer Systems

**Figure 6:**
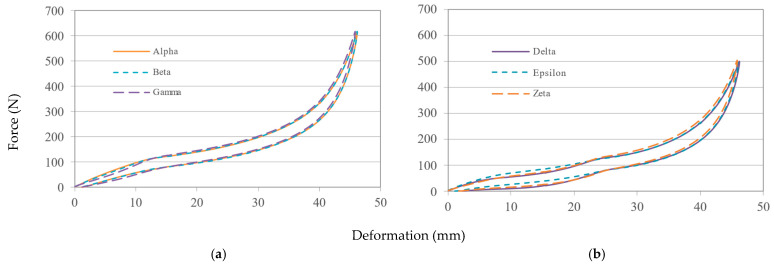
IFD curves depending on the arrangement of foam layers for: (**a**) Group X; (**b**) Group Y.

The IFD curves of Alpha, Beta and Gamma (Figure 6a) almost completely overlap over the entire deformation range, indicating a highly consistent compressive response despite different stacking sequences. All composites show a smooth, progressive increase in force with deformation, with no abrupt stiffness transitions. This confirms that layer order has only a minor influence on the global load-deflection behavior when S3, S8 and S11 are combined.

The IFD curves for Group Y (Figure 6b) also exhibit generally similar curve shapes, but with greater dispersion, especially in the low and medium deformation regions. This indicates a stronger dependence on layer sequencing. Comparable findings have been reported for layered foam cushions, where pressure redistribution and comfort-related performance were affected by layer configuration, surface/supporting layer interaction, and applied loading conditions [6,7,22].

Overall, Group Y shows lower terminal forces than Group X and a more pronounced sensitivity to stacking sequence, consistent with the higher damping and lower recovery previously observed. Group Y demonstrates a softer global response and greater configuration dependence across the low-to-medium deformation range. The substitution of conventional foam with LR reduces overall stiffness and shows greater variability in the IFD curves, particularly in the region most relevant to seated comfort. While Group X behaves as a mechanically stable system, Group Y allows more targeted tuning of surface compliance and progressive stiffness through layer ordering.

These preliminary results show that the mechanical behavior of multilayer foam systems intended for seating and lying applications is determined primarily by material selection rather than by layer arrangement alone. In systems composed of conventional and high-resilience PUR foams combined with latex rubber (Group X), the global response remained stable across different stacking sequences. It is interesting to note that in comparison to Antal et al. (2017)—where the lowest peak pressures were found precisely in the multilayer combination with the high–low–low density foam configuration [6]—that this matches our Gamma configuration in terms of the best support factor. Force–indentation behaviors were nearly identical across all configurations, as well as support factor, recovery, and hysteresis loss. This indicates that the PUR layers govern the system response and limit the influence of the latex layer. Rearranging layers, in such structured composites, does not meaningfully alter functional performance relevant to sitting or lying comfort.

Introducing low-resilience foam (Group Y) changed this behavior, although one of its intrinsic properties (Sf) differed only slightly from conventional PUR at the single-layer level. These systems show higher support factor values, but reduced recovery. Increased hysteresis loss values reflect greater energy dissipation and slower elastic response. This is in line with a study showing that mechanical response (hysteresis loss, surface firmness, bottoming resistance) strongly depends on chemical structure and molecular mobility [11]. Here, layer sequencing became relevant, especially when placing the LR foam on the surface provokes increased damping and reduced recovery, while positioning it deeper in the structure partially mitigated these effects, which is consistent with the results [22] where viscoelastic foam in the top layer provides surface softness but lowers sag factor, hysteresis loss, and stress relaxation.

The force–indentation curves confirm these trends. Group X behaved as a coherent system with a predictable progression in stiffness, while Group Y showed sensitivity to stacking order. Between the low and medium stress ranges, which are most relevant to contact pressures in sitting and lying positions, this proved particularly pronounced. This indicates that LR foams allow targeted tuning of surface compliance and damping but reduce mechanical robustness.

Together, the force–indentation curves, recovery values, and hysteresis loss results provide a useful experimental basis for future nonlinear viscoelastic FEM/FEA modeling of multilayer foam composites. Accurate numerical modeling of polymer foams requires experimentally determined constitutive parameters and validation against measured responses, particularly because flexible foams exhibit nonlinear, time-dependent, and viscoelastic behavior [8,10,12]. Accordingly, the experimental results obtained here provide a valuable reference dataset for future research focused on the development, calibration, and validation of a nonlinear viscoelastic FEM/FEA model for multilayer PUR and latex foam composites.

## 4. Conclusions

Considering only tested samples of selected types of foams, their qualities, physical and mechanical properties, the conclusions presented here can only serve as guidelines and trends in the mutual relationships of the observed composites. This research shows that the interrelationships among various commercial foams can be determined in a simpler, more accessible way, enabling selection of the optimal configuration for a particular purpose.

These preliminary results indicate that multilayer systems dominated by PUR foams tend to exhibit a predominantly elastic response with stable recovery and load-deflection behavior; whereas, the introduction of LR foam increases viscoelastic damping and yields a more configuration-dependent mechanical response.

The proposed hypotheses were confirmed. The findings indicate that the softest and most dissipative layer controls recovery, hysteresis, and perceived responsiveness, while higher-support layers have limited ability to compensate for its effects. Effective cushion design, therefore, requires prioritizing foam type selection, with layer arrangement serving as a secondary tool for fine-tuning surface feel and deformation behavior.

Referring to the limitations noted in the Introduction, this study, despite not including real pressure bodies or real-use scenarios, contributes to the understanding of multilayer foam behavior under standardized testing conditions. The preliminary results listed here may support future experimental planning and the easier selection of individual foam layers to improve elastic properties and potentially increase sitting and lying comfort.

The limited availability of specific raw data for commercial foams (such as compression modulus, hysteresis loss, recovery behavior, hardness, etc.) provides additional value to the results of this study, particularly because such data may be commercially sensitive and therefore difficult to access. As noted in previous research, material models require this type of foam-specific mechanical test data, for example for curve fitting, as essential inputs for FEA design calculations. The limitations of this study are the small sample size and the lack of more repeated measurements for the same type of composite. Also, since the cover materials are known to affect the strength of the foam, the results obtained may not be valid for seats or mattresses that have a fabric or leather cover in their final form.

Future research on multilayer composites should be planned with a realistically shaped pressure pad, so that the results can later be more reliably compared with objective measurements of human comfort.

## Figures and Tables

**Table 1:** Sub-samples markings, types, thickness, density and indentation hardness value of PUR and latex foams.

Sub-Sample Code	Foam Type	Thickness (mm)	Density (SD) ISO 845 [28] (kg/m^3^)	Indentation Hardness (SD) ISO 2439 [1], HB_(40%/30s)_ (N)	Commercial Coding	Foam Producer
S3-A1	Conv.	20.66	33.17 (0.09)	119.1 (8.75)	S 3534	Vapeks
S8-A2	HR	21.75	54.01 (0.10)	144.5 (1.32)	R 5235 Cellpur	Eurofoam
S11-A2	Latex	19.32	71.77 (2.28)	86.2 (3.55)	RG 65	Latexco
S12-A2	LR	20.58	53.92 (0.16)	48.5 (3.82)	V 5015	Vitafoam

Note: Conv.—conventional; HR—high-resilience; Latex—latex rubber; LR—low-resilience. The designations -A1 and -A2 refer to the thickness (~20 mm) and serial number of the sub-sample.

## Data Availability

All relevant data are included in this paper. The raw data supporting the conclusions of this article will be made available by the authors upon request.

## References

[1] (2008). Flexible Cellular Polymeric Materials—Determination of Hardness (Indentation Technique).

[2] (2025). Polymeric Materials, Cellular Flexible—Determination of Stress-Strain Characteristics in Compression—Part 1: Low-Density Materials.

[3] (2026). Standard Test Methods for Flexible Cellular Materials—Slab, Bonded, and Molded Urethane Foams.

[4] PFA (2022). InTouch Bulletin Series.

[5] Naddeo A., Cappetti N. (2021). Comfort Driven Design of Innovative Products: A Personalized Mattress Case Study. WORK J. Prev. Assess. Rehabil..

[6] Antal R.M., Horvath P.G., Dénes L. (2017). Body Pressure Distribution Analysis of Layered Foam Systems. Pro Ligno.

[7] Guan Y., Duan Y., Zhou P., Xie Y., Liu Q., Bao J. (2026). Enhanced Comfort and Biomechanical Performance of Ejection Seat Cushions via Optimal Double-Layer Foam Design. Front. Bioeng. Biotechnol..

[8] Fazekas B., Goda T.J. (2018). Determination of the Hyper-Viscoelastic Model Parameters of Open-Cell Polymer Foams and Rubber-like Materials with High Accuracy. Mater. Des..

[9] Mills N.J., Fitzgerald C., Gilchrist A., Verdejo R. (2003). Polymer Foams for Personal Protection: Cushions, Shoes and Helmets. Compos. Sci. Technol..

[10] Mills N.J. (2006). Finite Element Models for the Viscoelasticity of Open-Cell Polyurethane Foam. Cell. Polym..

[11] Scarfato P., Di Maio L., Incarnato L. (2017). Structure and Physical-Mechanical Properties Related to Comfort of Flexible Polyurethane Foams for Mattress and Effects of Artificial Weathering. Compos. B Eng..

[12] Elfarhani M., Mkaddem A., Alzahrani A.A., Bin Mahfouz A.S., Jarraya A., Haddar M. (2019). An Applied Model for Predicting Memory Effects of Flexible Polyurethane Foams. Multidiscip. Model. Mater. Struct..

[13] Demirel S., Ergun Tuna B. (2019). Evaluation of the Cyclic Fatigue Performance of Polyurethane Foam in Different Density and Category. Polym. Test..

[14] Abdullah M., Ramtani S., Yagoubi N. (2023). Mechanical Properties of Polyurethane Foam for Potential Application in the Prevention and Treatment of Pressure Ulcers. Results Eng..

[15] Dounis D.V., Wilkes G.L. (1997). Structure-Property Relationships of Flexible Polyurethane Foams. Polymer.

[16] Onaifo J.O., Otabor G.O., Onaiwu G.E., Edogun P.I., Ifijen I.H. (2025). Polyurethane Foam: Production Processes and Advanced Material Characterization. Trop. J. Chem..

[17] Demirel S., Ergun Tuna B. (2019). Constant-Fatigue Performance of Different Polyurethane Foams for Sitting Purposes. Kastamonu Univ. Orman Fak. Derg..

[18] Hilyard N.C., Hilyard N.C., Cunningham A. (1994). Hysteresis and Energy Loss in Flexible Polyurethane Foams. Low Density Cellular Plastics.

[19] Iyer D., Srivastava S. (2024). Facile Classification of Polyurethane Foam from Post-Consumer-Use Mattresses.

[20] Dwyer F.J. (1976). A Review of Factors Affecting Durability Characteristics of Flexible Urethane Foams. J. Cell. Plast..

[21] Silva P., Ribeiro D., Postolache O., Seabra E., Mendes J. (2024). Static Factors in Sitting Comfort: Seat Foam Properties, Temperature, and Contact Pressure. Appl. Sci..

[22] Moon J., Sinha T.K., Kwak S.B., Ha J.U., Oh J.S. (2020). Study on Seating Comfort of Polyurethane Multilayer Seat Cushions. Int. J. Automot. Technol..

[23] Paul G., Lee Y.J., Slattery P. (2020). Modelling of Multilayered Foams for Universal Seat Design. Advances in Transdisciplinary Engineering.

[24] Liu S., Siridhar G., Wang X. A Case Study of the Effects of Foam Properties and Support Surface on Seat Pressure Distribution. Proceedings of the Fourth International Comfort Congress.

[25] Ebe K., Griffin M.J. (2001). Factors Affecting Static Seat Cushion Comfort. Ergonomics.

[26] Hu L., Tor O., Shen L., Zhang J., Quin F., Yu X. (2020). Cushioning Capability Analysis of Seat Foundations Considering the Sitter’s Anthropometric Dimensions. Bioresources.

[27] Ferguson-Pell M.W. (1990). Seat Cushion Selection. J. Rehabil. Res. Dev..

[28] (2006). Cellular Plastics and Rubbers—Determination of Apparent Density.

[29] Vlaović Z., Vidoni N., Mihulja G., Miklečić J., Španić N., Klarić M. (2025). Exploring Elastic Properties of Flexible PUR and Latex Foams for Furniture. Proceedings of the 33rd International Conference on Wood Science and Technology (ICWST): Unleashing the Potential of Wood-Based Materials, Zagreb, Croatia, 4–5 December 2025.

[30] PFA (2011). Joint Industry Foam Standards and Guidelines.

